# Examining the implementation of the Linda Mama free maternity program in Kenya

**DOI:** 10.1002/hpm.3298

**Published:** 2021-08-11

**Authors:** Stacey Orangi, Angela Kairu, Joanne Ondera, Boniface Mbuthia, Augustina Koduah, Boniface Oyugi, Nirmala Ravishankar, Edwine Barasa

**Affiliations:** ^1^ Health Economics Research Unit (HERU) KEMRI‐Wellcome Trust Research Program Nairobi Kenya; ^2^ Independent Consultant Nairobi Kenya; ^3^ ThinkWell Nairobi Kenya; ^4^ Department of Pharmacy Practice and Clinical Pharmacy School of Pharmacy College of Health Sciences University of Ghana Accra Ghana; ^5^ Centre for Health Services Studies University of Kent Canterbury UK; ^6^ The University of Nairobi Kenyatta National Hospital Nairobi Kenya; ^7^ ThinkWell Washington DC USA; ^8^ Centre for Tropical Medicine and Global Health Nuffield Department of Medicine University of Oxford Oxford UK

**Keywords:** health policy, implementation, maternal health, process evaluation, user fees

## Abstract

**Background:**

In 2013, Kenya introduced a free maternity policy in all public healthcare facilities. In 2016, the Ministry of Health shifted responsibility for the program, now called *Linda Mama*, to the National Hospital Insurance Fund (NHIF) and expanded access beyond public sector. This study aimed to examine the implementation of the *Linda Mama* program.

**Methods:**

We conducted a mixed‐methods cross‐sectional study at the national level and in 20 purposively sampled facilities across five counties in Kenya. We collected data using in‐depth interviews (*n* = 104), administered patient‐exit questionnaires (*n* = 108), and carried out document reviews. Qualitative data were analysed using a framework approach while quantitative data were analysed descriptively.

**Results:**

*Linda Mama* was designed and resulted in improved accountability and expand benefits. In practice however, beneficiaries did not access some services that were part of the revised benefit package. Second, out of pocket payments were still being incurred by beneficiaries. Health facilities in most counties had lost financial autonomy and had no access to reimbursements from NHIF for services provided; but those with financial autonomy were able to boost facility revenue and enhance service delivery. Further, fund disbursements from NHIF were characterised by delays and unpredictability. Implementation experiences reveal that there was inadequate communication, claim processing challenges and reimbursement rates were deemed insufficient.

**Conclusions:**

Our findings show that there are challenges associated with the implementation of the *Linda Mama* program and highlights the need for process evaluations for programs to track implementation, ensure continuous learning, and provide opportunities for course correcting programs' implementation.

## 1 BACKGROUND

Globally, an estimated 295,000 maternal deaths occurred in 2017, resulting in a maternal mortality ratio (MMR) of 211 per 100,000 live births with sub‐Saharan Africa (SSA) accounting for 66% of these deaths.^1^ The 2014 Kenya Demographic Health Survey reported a MMR of 362,^2^ which is still unacceptably high. Countries have set out to reduce global MMR to less than 70 per 100,000 live births with no country having more than twice the global average by 2030.^3^, ^4^

Poor access to and low utilisation of skilled care during the antenatal, childbirth, and postnatal period contributes to high maternal deaths in Kenya.^5^ For example, only 62% of live births in the 2014 Kenya Demographic Health Survey were delivered by a skilled birth attendant and only 58% of mothers attended four or more antenatal visits.^2^

One key dimension of equitable access is affordability^6^ and therefore healthcare financing of maternal health is critical. The Kenyan health sector is financed from public, private, and donor sources accounting for 37%, 39.6%, and 23.4% of total health expenditure.^7^ Household out of pocket (OOP) payments account for a large proportion (26.1%) of total health expenditure.^7^ In 2018, 7.1% of Kenyan households incurred catastrophic health expenditures, resulting in 1 million Kenyans being pushed into poverty.^8^

Countries across Africa have undertaken health financing reforms to reduce OOP payments in order to increase utilisation of maternal health services, which in turn can reduce maternal deaths. For example, Morocco, Tanzania, and Senegal have removed user fees for deliveries and caesarean sections in public health facilities while Burundi and Ghana have this same policy but it extends to private sector.^9^, ^10^, ^11^ A free caesarean section policy has been instituted in all public health facilities in Mali and both the public and private not‐for‐profit health facilities in Benin.^12^, ^13^ In Uganda, there is a user fee removal policy in all public health facilities while in Zambia, the same policy extends to all rural facilities.^14^, ^15^ Burkina Faso, has universal care for all pregnant women in public and some private facilities.^16^

In Kenya, there have been various user fee reforms in relation to maternal health. In 2004, there was the abolition of user fees in all primary level facilities and an introduction of the 10/20 policy where a registration fee of Kenya Shillings (KES)10 and KES20 was charged in dispensaries and health centres respectively (approximately 2018 USD0.33 and USD0.67 respectively).^17^ Later in 2007, fees for deliveries in public healthcare facilities were removed. It is reported that there was a low adherence to the 10/20 policy and facilities still charged patients higher fees.^17^ This led to the abolition of the 10/20 policy in 2013 and an introduction of the free maternity policy resulting in removal of user fees for maternal healthcare in all public healthcare facilities, following a presidential declaration.^18^ In a distinct departure from the past, the national government started compensating health facilities for the lost revenue. An evaluation of this free maternity policy reported that although it led to an increase in utilisation of services there were several challenges.^19^ In October 2016, the Government of Kenya moved the management of the free maternity policy from the Ministry of Health to the NHIF and expanded service access under the programme to include private providers. The revised free maternity policy labelled “*Linda Mama*” is currently being implemented by the NHIF.^20^

*Linda Mama* is one of Kenya's pro‐poor policies intended to benefit the poor and vulnerable, thus revision of the free maternity policy was intended to reduce inequities in access to maternity services, and improve service access, accountability and operational efficiency of the program. However, from the Kenyan experience and that of other settings, actual implementation of policies are equally important as their design.^11^, ^17^, ^19^, ^21^, ^22^ This paper presents findings on a study to examine the implementation of the *Linda Mama* program in Kenya.

## 2 METHODS

### 2.1 Study setting

In 2010, Kenya transitioned from a centralised system of governance to a devolved system comprising a national government and 47 semi‐autonomous counties.^23^ National government retained policy development and regulatory functions, management of the national referral health facilities, capacity building, and technical assistance to counties; County governments on the other hand are responsible for service delivery and management of county health facilities.^24^ The public sector is organised hierarchically into 4 tiers (6 levels): (I) Community health services (level 1) (II) Primary care provided by dispensaries (level 2) and health centres (level 3) (III) County referral services including first referral sub‐county hospitals (level 4) and second referral county hospitals (level 5) (IV) National/tertiary referral hospitals (level 6).^24^

### 2.2 Study design

We conducted a mixed‐methods cross‐sectional study using qualitative and quantitative approaches at the national level and in five counties of Kenya. Data were collected between June and August 2019. We selected counties purposively in consultation with the NHIF.

Selected counties included two sites with Universal Health Coverage (UHC) initiatives. One was among the country's four UHC pilot sites implemented by the national government where user fees at public hospitals were removed; securing commodities through the Kenya Medical Supplies Authority was to be ensured coupled with conditional grants from the national government for lost revenue.^25^ In the public sector, this UHC pilot program had an overlap with the *Linda Mama* Program as its focus was on primary health care including immunisation, maternal and child health (MCH), family planning, TB, HIV, sexually transmitted diseases, and improved nutrition of pregnant women until the first five years of a child's life.^26^

The other had a county‐run UHC program in all public facilities in the county and covered inpatient services and outpatient services, but excluded some specialised services as well as family planning, maternal, neonatal and child services.^27^ The county‐run UHC program had no overlap with the *Linda Mama* program. These counties were selected because the NHIF was keen to understand *Linda Mama* implementation in both public and private facilities in the context where parallel initiatives were in place. Table 1 presents the demographics and health indicators of the study counties.

**TABLE 1:** County demographics and health indicators

Indicator	County A	County B	County C	County D	County E	Kenya
Total population (2019)^28^	1,453,787	268,002	987,653	885,771	1,157,873	47,564,296
Percentage share of urban population (2009)^29^	26	44	12	14	7	29.9
Percentage receiving antenatal care from a skilled provider (2014)^2^	98.2	96	98.0	98.5	91.6	95.5
Percentage health facility delivery (2014)^2^	52.6	42.1	53.3	46.5	38.6	61.2
Percentage delivery by a skilled provider (2014)^2^	52.3	43.8	54.6	46.8	40.3	61.8
Health personnel in the public sector (per 100,000 people) (2013)^30^, ^31^, ^32^, ^33^, ^34^						
Nurses	30	149	43	38	29	55
Doctors	4	30	4	3	6	10
Clinical officers	17	36	22	20	9	21
Number of health facilities (2015)^30^, ^31^, ^32^, ^33^, ^34^						
Public	10	38	180	139	100	4929
Non‐governmental	9	0	3	3	8	347
Faith based	14	11	29	17	34	1081
Private	127	5	46	46	21	3797

Across the five counties, 20 healthcare facilities were purposively sampled for data collection. These included one public county referral hospital, one public sub‐county hospital, one public health centre, and one faith‐based hospital/health centre in each of the sampled counties.

### 2.3 Conceptual framework

As a process evaluation,^35^, ^36^, ^37^ our research sought to examine how a program was implemented and to generate evidence that could explain observed policy outcomes. We also sought to highlight opportunities for course‐corrections in implementation that may help the program attain its intended outcomes.^35^, ^36^, ^37^ In this study, we assessed the emergence of the *Linda Mama* program, its implementation fidelity, and implementation experiences of various actors. An assessment of the emergence of the program examined factors that led the previous free maternity policy to morph into the *Linda Mama* program. Assessing the fidelity, entailed comparing what the intended program design was (‘de jure’ policy) against what was being implemented in practice (‘de facto’ policy).^35^, ^37^ We examined implementation fidelity across key dimensions of the program namely the program beneficiaries, the benefit package, financing arrangements, and facility contracting for quality. We also explored the experiences of various actors involved in the implementation of the policy.

### 2.4 Participant selection and data collection

#### 2.4.1 Qualitative component of the study

After obtaining written consent from participants, we collected qualitative data using in‐depth interviews and document reviews. We conducted interviews (*n* = 104) with purposely selected participants drawn from the national and county level who had knowledge on the *Linda Mama* program either because of their roles and/or experience in implementing the program. Data collection was discontinued upon data saturation. At the national level, participants were drawn from Ministry of Health, NHIF national and regional branch officials, private sector umbrella organisations, and developmental organisations supporting health financing interventions. At the county level, we selected participants from the county health management teams (CHMT) and healthcare facilities. Table 2 outlines the distribution of study participants.

**TABLE 2:** Number and type of study participants

Respondents	National	County A	County B	County C	County D	County E
National level in‐depth interviewees						
Ministry of health officials	5					
Private umbrella organisations and development organisations	7					
NHIF officials	3	1	1	1	1	1
County level in‐depth interviewees						
Chief Officer Health/County Director of Health/RMNCH Coordinator/County Accountant/County health administrator		2	2	3	2	3
Facility level in‐depth interviewees						
Facility/Nurse in‐charges		4	4	4	4	3
Maternity/ANC in‐charge		6	2	5	3	3
Front line workers in maternal health		2	4	3	3	3
Facility NHIF clerks		4	4	4	4	3
Patient exit interviewees						
Antenatal care patients		8	8	6	8	8
Delivery patients		12	11	12	11	8
Postnatal care patients		3	3	3	3	4

Abbreviation: NHIF, National Hospital Insurance Fund.

We reviewed policy documents including circulars or policy communications from NHIF to the facilities and the implementation manual.

#### 2.4.2 Quantitative component of the study

We collected quantitative data using structured questionnaires and data abstraction tools. We used a researcher administered structured questionnaire to carry out patient‐exit interviews to determine the level of OOP costs paid by *Linda Mama* beneficiaries to access maternal services: Participants were consented prior to this. A sample size was determined based on a 95% confidence level, 10% margin of error and assuming 50% reported OOP payment by *Linda Mama* beneficiaries in the population.^38^, ^39^ We randomly selected patients seeking maternal care (*n* = 108): antenatal, delivery, and postnatal care from the selected facilities. Table 3 outlines the demographic characteristics of patient‐exit interview participants.

**TABLE 3:** Characteristics of patients that participated in patient exit interviews

Characteristics	Observations (*n* = 108)	Proportion (95% CI)
Mean age in years	108	26.6 (24.8–28.3)
Highest level of education		
No education	8	7.4% (3.7–14.3)
Primary level	43	39.8% (30.9–49.5)
Secondary level	40	37.0% (28.3–46.7)
Tertiary level	17	15.7% (9.9–24.0)
Marital status		
Married	91	84.3% (76.0–90.0)
Divorced	0	0%
Separated	2	1.9% (0.5–7.3)
Widowed	0	0%
Unmarried	15	13.9% (8.5–21.9)
Employment status		
Full‐time employed	8	7.4% (3.7–14.3)
Part‐time employed	2	1.9% (0.5–7.3)
Unemployed	62	57.4% (47.8–66.5)
Self‐employed	31	28.7% (20.9–38.1)
Student	5	4.6% (1.9–10.8)
Mean number of children	108	2.1 (1.7–2.4)
Registered with *Linda Mama* program		
Yes	78	72.2% (62.9–80.0)
No	30	27.8% (20.0–37.1)
Type of visit		
Antenatal care visit	38	35.2% (26.7–44.8)
Delivery visit	54	50.0% (40.5–59.5)
Postnatal care visit	16	14.8% (9.2–23.0)

We also assessed the structural quality of care offered in the selected healthcare facilities by collecting data on the availability of tracer medicines and medical equipment that are essential for MCH. Review of administrative data from the facility records and NHIF claims management system was done to examine funding flows for the *Linda Mama* program and determine patterns of claims versus reimbursements.

### 2.5 Data analysis

Audio recordings were transcribed verbatim in Microsoft Word and those that were in Kiswahili were translated to English. A framework approach was used for the qualitative analysis; it entails familiarisation, identifying a thematic framework, coding, charting, and interpretation of results.^40^ Familiarisation of the data, through listening to the audios and reading the transcripts, ensured transcription and translation accuracy. Following verification, coding of transcripts was done based on a thematic framework developed and agreed upon by the investigators that was derived from the conceptual framework. After indexing of the transcripts and ensuring new emerging themes were captured, charting was done. Charting involved summarising the findings of the transcripts based on the identified themes and identifying illustrative quotes. Data analysis was completed by identifying associations between the themes and providing explanations relevant to the objective of the study.

Quantitative data was entered in Microsoft Excel then imported to Stata Version 15.0 for data cleaning and analysis. Data cleaning was based on logic checks and frequency distributions. Descriptive analysis was done using frequency distributions, measures of central tendency and dispersion, using means and 95% confidence intervals or medians and interquartile ranges, as appropriate.

## 3 RESULTS

The following section presents study results, describing the emergence of the *Linda Mama* program, its implementation fidelity, and finally the implementation experiences from the various actors.

### 3.1 Emergence of the *Linda Mama* program

Findings from the interviews reveal that the *Linda Mama* program was developed to address challenges identified with the previous free maternity program. These challenges were (1) poor accountability mechanisms within Ministry of Health as it was not able to verify actual versus fictitiously reported deliveries (2) a narrow benefit package that only covered deliveries, (3) beneficiaries having restricted access to free maternity services only through public healthcare facilities, and (4) duplication of payments for deliveries by Ministry of Health and NHIF for mothers who were NHIF members.

“The earlier free maternity program was a direct transfer of *funds* without any mechanism to verify health facility claims…We wanted to introduce that monitoring aspect in the revised free maternity program (*Linda Mama*). Also, the previous program did not include antenatal care and postnatal care which we wanted to include in *Linda Mama*.” **Representative, Developmental partner organization**

“The other challenge was duplication. While the Ministry of Health reimbursed healthcare facilities for deliveries, NHIF reimbursed the same facilities for deliveries too. There was also a need to facilitate access to maternity services from faith‐based and private facilities.” **Senior manager, NHIF**

NHIF, the country's sole public insurance agency, was deemed suitable for management of the program. It also had existing structures such as local branches throughout the country, claims processing mechanisms and capacity, contracting mechanisms with the private sector.

### 3.2 Implementation fidelity

#### 3.2.1 Program beneficiaries

In some counties, newborns were excluded from benefiting from *Linda Mama*

According to the implementation manual, the intended beneficiaries of the *Linda Mama* program were Kenyan pregnant women and their newborns for one year. However, in some counties, newborns were not considered beneficiaries of the program, reflecting some misunderstanding about their inclusion and how to make a claim for reimbursements for these services.

“If the mother delivers and develops complications, or we need to admit the newborn, *Linda Mama* does not pay for the baby…*Linda Mama* just caters for the mother and not the baby” **Maternity‐in‐charge, faith‐based facility, county A**

#### 3.2.2 Benefit package

*Linda Mama* beneficiaries did not access some services that were part of the *Linda Mama* benefit package

De jure, the benefit package included antenatal care, delivery services, postnatal care, emergency referrals, conditions and complications during pregnancy, and care for the newborns per the national guidelines (Table 4). However, respondents across the sampled counties reported that the benefit package in practice did not cover some of the key services included. Services excluded in practice were newborn care, outpatient complications for the mother, ultrasounds, family planning as part of postnatal care, immunisation, medical abortions, Anti‐D medications, and transport for emergency referrals.

"Anti‐D is also not covered. We have rhesus positive mothers and it's not covered.” **Medical Superintendent, public hospital 1, county D**

**TABLE 4:** Benefit package and reimbursement rates (de jure)

		Reimbursement rates according to the *Linda Mama* implementation manual					
**Services**	**Benefit package according to the *Linda mama* implementation manual**	**Public primary care facilities (Tier 2)**	**Public primary and secondary referral facilities (Tier 3)**	**Public tertiary referral facilities (Tier 4)**	**Private/Faith based primary care facilities (Tier 2)**	**Private/Faith based primary and secondary referral facilities (Tier 3)**	Notes
Antenatal care	ANC profile, preventive services, prevention of mother to child transmission of HIV	KES 600 (USD 6)	KES 1000 (USD 10)	KES 1000 (USD 10)	KES 1000 (USD 10)	KES 1000 (USD 10)	Reimbursement for ANC‐1^st^ visit
		KES 300 (USD 3)	KES 300 (USD 3)	KES 500 (USD 5)	KES 500 (USD 5)	KES 500 (USD 5)	Reimbursement for ANC‐subsequent 3 visits
Delivery	Skilled delivery (including caesarean section), neonatal care including costs related to preterm births	KES 2500 (USD 25)	KES 5000 (USD 50)	KES 17,000 (USD 170)	KES 2500 (USD 25)	KES 6000 (USD 60)	Reimbursement for normal delivery
		___	KES 5000 (USD 50)	KES 17,000 (USD 170)	___	KES 17,000 (USD 170)	Reimbursement for caesarean section delivery
Postnatal care	Vitamins, family planning services, screening, immunisation, and early infant diagnosis of HIV	KES 250 (USD 2.5)	KES 250 (USD 2.5)	KES 250 (USD 2.5)	KES 250 (USD 2.5)	KES 250 (USD 2.5)	Reimbursement for PNC and new‐born care (each of the 4 visits)
Emergency referrals	Ambulance services						
Conditions and complications during pregnancy	Outpatient and inpatient treatment						
^a^Care for the infant	Outpatient services including treatment and child welfare clinics, and inpatient services						

*Note*. Source: Linda Mama implementation manual and circulars sent to facilities from the NHIF.

Abbreviation: NHIF, National Hospital Insurance Fund.

^a^ Care of the infant is within the 1‐year period that the mother is in the program.

#### 3.2.3 Financing arrangements

##### *Linda Mama* beneficiaries incurred some OOP expenditure to access maternal services

The *Linda Mama* program intended to eliminate OOP payments for accessing maternal services. In Counties A, C, D, and E, *Linda Mama* funds were the main source of reimbursement for maternal care services amongst the sampled patients. However, exit interviews revealed that 9%, 14%, 19%, 45%, and 52% of the sampled mothers in county B, D, C, E, and A respectively supplemented with OOP payments.

**FIGURE 1:**
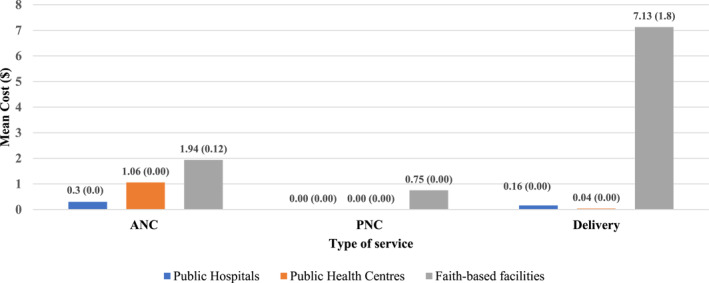
Out of pocket medical costs incurred at the facility (mean cost [median cost])

The mean reported OOP costs during an ANC visit ranged from $0.3 (median = $0) in public hospitals to $1.94 (median = $0.12) in faith‐based facilities; items paid for included ultrasounds, drugs, and photocopy costs. For PNC visits, no OOP costs were incurred at the public facilities, however, the mean OOP cost in faith‐based facilities was $0.75 (median = $0) and was mainly drug costs. Lastly, mean OOP costs for deliveries ranged from $0.04 (median = $0) in public health centres to $7.13 (median = $1.8) in faith‐based facilities. Items paid for during delivery visits included drugs for the newborn, basins, cotton wool, tissues, photocopy, chlorhexidine, cannula, NG‐tube costs, registration costs, and for *mama* kits (care packages). Details of the OOP medical costs incurred at the facilities are illustrated in Figure 1.

There was a substantial number of patients who had to incur medical costs outside the facility for either drugs or other medical items due to their unavailability at the facility. This ranged from 3%–24% of the sampled mothers in public health facilities and 0%–27% of the sampled mothers in faith‐based facilities.

“Several Kenyans who deserve *Linda Mama* are not getting it because essential commodities are not available. When you tell women to go and get a bucket, spoon, consumables, it ceases to be a free service” **Representative, developmental partner organization**

##### Some public healthcare facilities could not access funds from *Linda Mama* reimbursements by the NHIF

**FIGURE 2:**
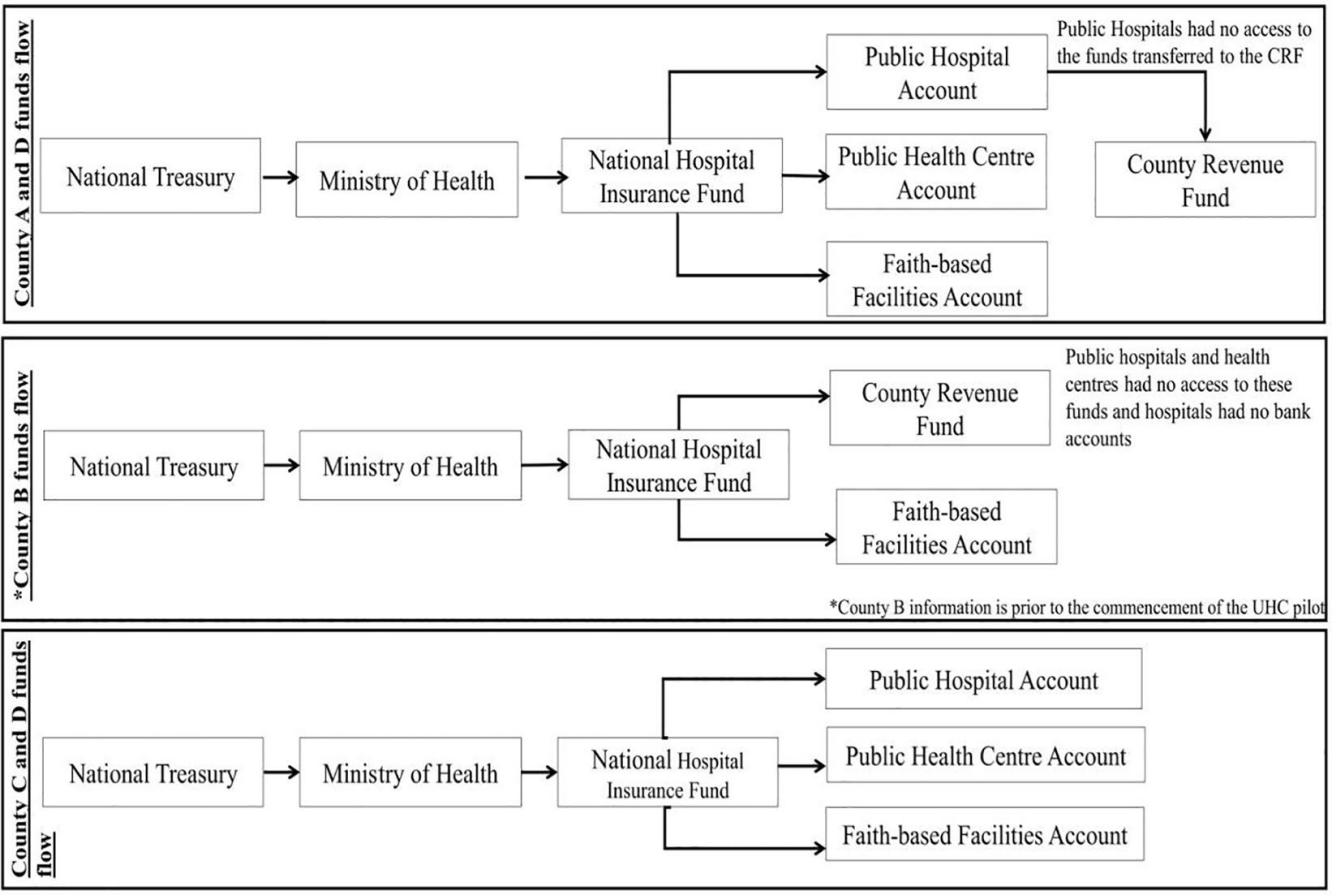
Funding flows of *Linda Mama* funds to facilities (de facto)

*Linda Mama* is funded using tax funds and according to the program, funds were to flow from NHIF directly to the individual healthcare facility's bank account. In actual practice, private and faith‐based healthcare facilities received the funds directly to their bank accounts. For the public sector de facto flow of funds varied as illustrated in Figure 2.

The main reported challenge with the funding flow arrangement is that in some counties, the funds are redirected to the county revenue fund (CRF) account controlled by the county and the funds not remitted back to the healthcare facilities. Under this arrangement, healthcare facilities lost their financial autonomy, and county departments of health (CDOH) were responsible for procuring supplies, drugs, and paying for all operational costs for the healthcare facilities.

The lack of autonomy affected public healthcare facilities' ability to access the funds and procure necessary hardware or hire staff to lodge claims, which would enable them to generate more revenue from the program. Additionally, it affected the quality of care offered due to the inability to purchase essential supplies.

"If the county could reinvest the funds to health facilities, the facilities would be able to hire more casual workers who can do registration and claim processing, buy computers, install Internet services or buy a mobile phone for that facility to do this process.” **Senior manager, NHIF**

"There are things which we may miss that are very basic to assist a mother during delivery. When we don't have access to the funds, we are unable to purchase these essential supplies.” **Nursing officer in charge, public hospital 2, county D**

Facilities that receive funds for *Linda Mama* services and do not need to redirect it to the CRF account, had the freedom to use the funds according to their priorities. This may not necessarily be in MCH but needed to be based on work plans and sought approvals. This positively strengthened the health system in general and ensured effective service delivery for *Linda Mama* services.

"Yes, in County C, they have the freedom to spend all the money collected by the hospital, 100%, based on their needs and priorities. And they must follow regulations.**” County Health Administrator, county C**

Funding disbursement by the NHIF to healthcare facilities was associated with delays.

**FIGURE 3:**
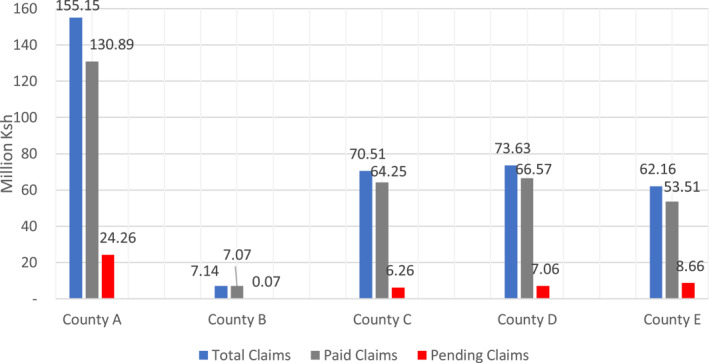
Claims summary per county. July 2018‐March 2019

According to the implementation manual, NHIF was to ensure timely payment of providers for *Linda Mama* services rendered. This was specified to be within 30 days of receiving the invoices. In practice, however, healthcare facilities reported delays in receiving payments from NHIF; the timing and amount to be reimbursed was also unpredictable. This resulted in pending claims to facilities which ranged from 1%–16% as illustrated in Figure 3.

"There is a big challenge with the flow of funds because our hospitals are owed a lot of money by NHIF and there are delays in the disbursement of these funds. The amount that we expect to be disbursed to us is also not predictable.” **County health administrator, county C**

Unpredictability, and delays in disbursement of funds to healthcare facilities, negatively affected service delivery due to difficulties in financing for essential supplies.

“The delays affect service delivery because a hospital is just like a hotel…A mother comes, uses my supplies, and goes… But I have not been paid, it depletes my supply and that results in poor service” **County health administrator, county C**

The delays in disbursement of funds by NHIF to healthcare facilities were partly blamed on delays by Ministry of Health to disburse *Linda Mama* funds to NHIF. For instance, in the financial year 2018/2019, the government did not release all the *Linda Mama* funds as expected, therefore, NHIF was off the budget target by 14%.^41^

"The other challenge that we have is that it takes a while before Ministry of Health gives us money and this is a managed scheme. If we don't have money, we stop there, and we wait until funds come. We do not pay any facility until when we receive funds, then we start releasing money.” **Senior manager, NHIF**

#### 3.2.4 Facility contracting

##### Availability of essential medical supplies varied among sampled healthcare facilities

NHIF was expected to contract healthcare facilities with the structural capacity to provide the services in the *Linda Mama* benefit package. Essential medicines were available in almost all the sampled facilities on the day of data collection, except misoprostol which was only available in 55% (*n* = 11) of the facilities. Uninterrupted oxygen supply in labour ward was available in approximately 65% (*n* = 13) of the facilities. One facility did not have soap and running water/alcohol rub in the childbirth and neonatal ward. This is reported in Table 5.

**TABLE 5:** Structural quality: availability of essential medicines and supplies

Essential medicines and supplies	Available today	Available in the last 90 days
Penicillin	95% (*n* = 19)	90% (*n* = 18)
Metronidazole	95% (*n* = 19)	90% (*n* = 18)
Gentamicin	95% (*n* = 19)	85% (*n* = 17)
Oxytocin	95% (*n* = 19)	95% (*n* = 19)
Misoprostol	55% (*n* = 11)	55% (*n* = 11)
Functional blood pressure machine	100% (*n* = 20)	
Functional bag and mask (two neonatal mask sizes)	100% (*n* = 20)	
Uninterrupted oxygen supply in childbirth	65% (*n* = 13)	
Uninterrupted oxygen supply in neonatal ward (10 facilities)	60% (*n* = 6)	
Uninterrupted oxygen supply in paediatric ward (10 facilities)	60% (*n* = 6)	
Soap and running water/alcohol rub in childbirth	95% (*n* = 19)	
Soap and running water/alcohol rub in neonatal ward (10 facilities)	90% (*n* = 9)	
Soap and running water/alcohol rub in paediatric ward (10 facilities)	100% (*n* = 10)	

### 3.3 Implementation experience

#### 3.3.1 Communication

County officials and healthcare facilities administrations received official communication on the program from NHIF branch offices through circulars and verbal communication. However, in Counties A and E, there was inadequate sensitisation and a lack of proper cascade of information to all county health officials and sub‐county teams. The county officials, although informed, were not sufficiently equipped to support the healthcare facilities with the challenges faced within the program; NHIF was dealing with the facilities directly.

In the UHC pilot county, public healthcare facilities had not been receiving reimbursement for *Linda Mama* since the UHC pilot program began. There was a lack of clarity at a county level, in the public and private healthcare facilities on whether the program was to continue after the rollout of the UHC pilot. Ministry of Health and NHIF reported that the program should be halted in public healthcare facilities during the pilot, however, these counties did not receive official communication.

"We are no longer reimbursing *Linda Mama* for the public health facilities in the UHC pilot counties because they have been given money for the inputs that they require… The only people who are claiming then would be the private and the NGO's in those pilot counties because they aren't under the UHC program currently.” **Ministry of Health official**

“I asked him (Ministry of Health official) what the effect of *Linda Mama* is since now there is money coming from national Government (UHC fund) … He told us that for *Linda Mama* we cannot claim but there is no official letter to that effect**.” Medical Superintendent, public hospital 1, county B**

There was a lack of information among the mothers in the community on the availability of *Linda Mama*, and unlike the previous free maternity policy, mothers had to register to access *Linda Mama* services. The majority of the interviewed mothers (71%) were made aware of the program at the facility level during their ANC or delivery visits. This suggests that those who do not access services at health facilities, lack information.

“At a community level, I think some of them are not aware of *Linda Mama*.” **Maternity in charge, faith‐based facility, county A**

#### 3.3.2 Healthcare providers felt that the *Linda Mama* reimbursement rates were insufficient

According to the policy, healthcare facilities were to be reimbursed for antenatal, delivery, and postnatal care through case‐based rates as indicated in Table 4. Inpatient services for the mothers (except deliveries) were to be paid for on a fee‐for‐service basis, based on agreed‐upon rebates with NHIF.

The respondents reported that the payment rates were insufficient to cover the costs of services provided and that reimbursement rates for deliveries were much higher in the other NHIF schemes.

“Private and faith‐based health facilities say the amount is low especially for caesarean section. Public healthcare facilities claim that the amount is low and question why they are paid the same rate regardless of the type of delivery (caesarean or normal delivery)” **Senior manager, NHIF**

“If you look at the amount reimbursed it is a bit less compared to NHIF national scheme reimbursement. At a facility level, we strain in terms of resources.” **Maternity in charge, faith‐based health centre, county A**

As a result of the perceived insufficient payment rates, most private and faith‐based facilities did not accept the contract with NHIF to offer *Linda Mama* services, limiting access. Further, patients across all types of facilities incurred OOP payments for some services.

“Very few private providers signed up for *Linda Mama* because private facilities are for profit. The rates that *Linda Mama* pays are perceived to be low.” **Senior NHIF manager.**

“Pregnant women don't always deliver in the facilities that they seek other pregnancy related care. If mothers don't deliver here, but spend some time here and consume drugs etc., I cannot claim from the *Linda Mama* package. Then now you want to refer but there is a bill that *Linda Mama* cannot foot, then the mother probably would foot the bill.” **Maternity in‐charge, faith‐based health centre, county A**

#### 3.3.3 Challenges with the claims process

Several challenges were identified with the *Linda Mama* claims process. First, there was inadequate training on how to make claims. This was further compounded by high staff turnover and a shortage of staff with no focal person to lodge claims, especially in lower‐level facilities. Second, public healthcare facilities lacked necessary hardware to lodge claims, the online e‐claim system had several system outages, and there were disruptions when facilities needed to switch between the manual and online system. Third, there were challenges processing claims for patients who lacked identification documents. Lastly, there was a lack of motivation among the staff to lodge claims, especially in cases where the facilities had no access to the reimbursements.

“We have system challenges as a county in the facilities. You would find that the health facilities would require a photocopy machine, but we don't have it. So, we keep telling them in their next claim, make sure to budget for photocopy machines… Another challenge is staff turnover and shortage. There is only one nurse who is in that dispensary…We have not empowered the facilities with skills (to claim), the supervisors, and the facilities to know how to mentor and build their capacity” **County director of health, county E**

“You find that we will use the e‐claim system then later the NHIF would ask us to submit manual claims too which is like repeating the same job and causes delays… But the thing that affects registering them is network and lack of airtime.” **NHIF clerk, public hospital 1, county E**

Challenges with the claims processing resulted in the loss of facility revenue and as a result a strain in essential resources.

In some of the health facilities, the claims process was made easier by having a focal NHIF clerk for processing the *Linda Mama* claims and ensuring all the healthcare workers had the knowledge of the claim process and requirements.

“We employed our NHIF clerks and it has boosted our revenue because they support health facilities to claim from the NHIF.” **Medical superintendent, public hospital 1, county D**

#### 3.3.4 Distance and associated transport cost were a barrier to access

In Counties B and E, the distance to facilities and transport costs was reported to be a barrier of access to care.

"The county is vast, so they have to walk long distances to reach the facility for *Linda Mama* services. Transport and poor road infrastructure are a challenge. If I take that boda‐boda [motorcycle] it will cost me KES500 to the dispensary and back.” **County director of health, county E**

## 4 DISCUSSION

Recognising that the implementation of policy is as important as its design, this study set out to determine the emergence of the *Linda Mama* policy, evaluate its implementation fidelity, and explore variations in the implementation experiences of various actors.

Our study revealed that the re‐formulation of the free maternity program was aimed at addressing challenges that were reported with the previous free maternity policy.^19^ These included poor accountability mechanisms within the Ministry of Health, narrow benefit package that only covered deliveries, limited access only through the public sector, and duplication of payments by Ministry of Health and NHIF.

Our findings show that although the program has expanded its benefit package and access to maternal health services, some challenges persist. First, *Linda Mama* beneficiaries did not enjoy the full set of services that they were entitled to according to the benefit package. They faced challenges accessing newborn care, outpatient treatment for complications, ultrasounds, medical abortions, Anti‐D medications, and transport for emergency referrals. They were also often told that family planning and immunisation were not included in the benefit package because they are offered through vertical donor‐funded programs. These vertical programs were however not available in some of the private and faith‐based facilities that offer *Linda Mama* services, limiting access. This is consistent with evidence that suggests that individuals enrolled to NHIF in Kenya do not receive the full entitlement outlined in the benefit package.^42^ Inadequate communication and engagement of implementers and beneficiaries after the reformulation of the policy could have contributed to this.

Undoubtedly, to ensure reforms are enforced in healthcare facilities, effective communication and engagement strategies are necessary.^11^ Lack of this, as evidenced in the implementation of *Linda Mama*, could result in the exclusion of some essential services outlined in the benefit package during service provision, raising equity concerns. Additionally, the perceived low payment rates could have created perverse incentives to healthcare providers to withhold provision of free services. Similar challenges with inadequate communication and understanding of the benefit package for free maternity programs have been reported in Ghana, Burkina Faso, Morocco, and Benin.^13^, ^43^, ^44^ For instance, Burkina Faso's benefit package included newborn care and post‐abortive care while ultrasounds and antimalaria drugs were included in Ghana, yet there was no free service provision for them.^13^, ^45^

Second, although the *Linda Mama* program was aimed at extending financial risk protection, there were instances where patients paid OOP either because of fees charged at healthcare facilities or they were asked to purchase items out of the facilities due to their unavailability. This could be the result of the aforementioned perverse incentives created by the perception that the payment rates are low, driving healthcare providers to charge for the costs incurred during service provision not covered by the reimbursements. Similar findings have been reported in Ghana, Mali, and Laos where reimbursements did not sufficiently cover the expenses for maternal health services resulting in the ineffective implementation of exemption policies and patients incurring OOP payments.^13^, ^46^, ^47^ Conversely in Burkina Faso, the actual expenses of maternal health services were reimbursed in full by their Ministry of Health.^48^ While providers in this setting could not cite insufficient reimbursements, OOP payments were still reported, although far less frequent than other SSA countries.^48^ In Benin, case‐based reimbursement was found to overpay the hospitals for caesarean sections.^13^ These excess funds although positively used by managers to improve service delivery, negatively incentivised providers to conduct avoidable caesarean sections and OOP payments were still incurred in some cases.^13^

Lastly, there was varying availability of essential medical supplies in the sampled healthcare facilities. This could be a result of the funding disbursement challenges that healthcare facilities faced which inhibited them from budgeting and procurement of essential supplies. For example, hospitals in most of the counties did not have the autonomy to spend *Linda Mama* funds. While the Kenyan public finance management act requires that counties operate one CRF account, the law also provides for county governments to develop bylaws that give financial autonomy to hospitals.^49^ The latter was successfully implemented by few counties.

On the other hand, under this program public primary level healthcare facilities were for the first time contracted by NHIF and required to lodge claims, a process unfamiliar to them. This coupled with other claims processing challenges disadvantaged them from receiving reimbursements under the program albeit offering free maternity services. Additionally, *Linda Mama* reimbursements were frequently delayed and unpredictable in timing and amounts paid from NHIF to healthcare facilities. Evidence suggests that for Kenyan hospitals, provider autonomy is important because reduced autonomy has been associated with delays in procurement of essential supplies, reduced staff motivation, and weakened external accountability.^49^ However, even with provider autonomy, inadequate and delayed payments can affect staff motivation, compromise quality of care, and cause withholding of free services, as was the case in Ghana and Nigeria.^50^, ^51^

The varying availability levels of essential supplies could also be because of the inadequate quality monitoring systems from both CDOH and NHIF. It has been previously reported that the limited number of NHIF officials weakens frequent quality monitoring systems to healthcare facilities.^42^ Our findings mirror those reported in Laos and Ghana where the quality of care and specifically the availability of essential supplies and equipment were impeded by insufficient spending on health and delayed payments respectively as well as an insufficient ability to provide supervision and technical assistance to healthcare facilities, as in the case of Laos.^45^, ^47^

The strength of the study is that it included a wide range of stakeholders at both the national, sub‐national, and facility level. The first limitation of this study is that the findings may not be generalisable. Second, due to the cross‐sectional nature of this study, we were unable to account for temporal variations. Third, this study excluded private‐for‐profit facilities due to a difficulty in gaining access during data collection; as a result, the reported findings are biased towards implementation of the program in public and private‐not‐for‐profit facilities. Further, the study did not look at utilisation and user level outcomes as this was examined elsewhere.^52^ We suggest that future studies can combine process evaluation of such programs with the utilisation trends over time. However, the study contributes to the literature on implementation experiences and fidelity of health financing reforms in similar low‐and middle‐income countries.

## 5 RECOMMENDATIONS

Drawing from the findings of this study, we make the following recommendations to strengthen the implementation of the *Linda Mama* free maternity program.

First, communication of the program to health facilities and beneficiaries should be improved. Ministry of Health should provide better clarity on how the program should be implemented in the UHC pilot counties and eventually during the country‐wide scale‐up. In addition to formal written communication such as circulars that would ensure better clarity on the benefit package, county health forums could be used as a platform for engagement between the county implementers and NHIF. Efforts to bridge the information gap on *Linda Mama* among the beneficiaries should be made by ensuring regular health talks, accompanied by effective information, education, and communication material displayed in healthcare facilities. The use of county community health teams as well as increased and regular NHIF campaigns for communities on *Linda Mama* should be utilised. These efforts should be accompanied by targeted messages to teenagers.

Second, existing financial arrangements for the program must be improved on. For instance, counties should work on the bottlenecks in the *Linda Mama* funding flow to healthcare facilities by developing a legal framework that would support healthcare facilities to retain the revenue that they generate. NHIF should address the delays in *Linda Mama* fund disbursements and predictability of payments. Further, Ministry of Health should address funding disbursement delays from them to NHIF. It is also important that NHIF and Ministry of Health review and engage healthcare providers on the program's provider payment rates.

Third, Ministry of Health and NHIF should revise the *Linda Mama* benefit package to ensure that it comprehensively covers all essential services for pregnant women, mothers, and newborns.

Fourth, claims processing challenges should be improved on in the following ways: 1) ensure healthcare facilities have the necessary hardware required to lodge claims 2) identification of training gaps in claims processing followed by training of county and sub‐county health officials and re‐training of appropriate and adequate numbers of healthcare providers; this would enable the county and sub‐county officials to efficiently support healthcare facilities on claims processing moving forward 3) ensure stability and proper functioning of the NHIF claim system 4) appointments of NHIF focal persons in the healthcare facilities to increase facility revenue, where feasible, and 5) in instances where power, internet access, or the required hardware would be inaccessible, county officials should work with those healthcare facilities to identify nearby facilities that have these resources and leverage on them.

Fifth, counties should monitor healthcare facilities to ensure no user fees are charged to women seeking *Linda Mama* services. This can be coupled with using citizen feedback to monitor compliance.

Sixth, NHIF should improve awareness among all healthcare workers on existing criteria of use of alternative documents in place of the patient identification documents, where unavailable, for claims submission. Alternatives such as birth certificates, letters from the chief should also be explored and clear exemption criteria should be outlined when the above documents cannot be obtained.

Lastly, the quality of services offered under the *Linda Mama* program must be improved on. Counties should ensure that there is an adequate number of human resource capacity coupled with an increase in MCH infrastructure. Additionally, the CDOH as well as NHIF should ensure regular support visits while ensuring availability of all essential medical supplies.

## 6 CONCLUSION

The *Linda Mama* free maternity program was introduced to protect the poor and vulnerable and improve delivery of maternal services. Our study findings show that there are challenges associated with its implementation, some of which are consistent with experiences from other low‐and middle‐income settings. The study points out existing inter‐county variations and demonstrated the value of tracking implementation that can make course corrections in the program possible.

## CONFLICTS OF INTEREST

The authors declare that they have no competing interests.

## AUTHOR CONTRIBUTIONS

Edwine Barasa, Boniface Mbuthia, Joanne Ondera, and Nirmala Ravishankar conceptualised the study. The interview guide was developed by Stacey Orangi, Boniface Oyugi, Joanne Ondera, Augustina Koduah, Nirmala Ravishankar, and Edwine Barasa. Data was collected by Stacey Orangi, Angela Kairu, and Boniface Oyugi. Stacey Orangi developed the coding tree which was reviewed by Augustina Koduah and Edwine Barasa. Coding, charting and mapping were conducted by Stacey Orangi with Augustina Koduah and Edwine Barasa contributing to the interpretation of the findings. The initial manuscript was drafted by Stacey Orangi which was subsequently revised for important intellectual content by all authors. All authors read and approved the final manuscript.

## ETHICAL STATEMENT

Ethics approval to conduct the study was obtained from the Kenya Medical Research Institute/Scientific and Ethics Research Unit (KEMRI/SERU/CGMR‐C/132/3735). Further, approvals were sought from the Council of Governors, National Commission for Science, Technology and Innovation, the respective county departments of health, and the health facilities management. Signed written informed consent was sought from each of the study participants prior to data collection.

## CONSENT FOR PUBLICATION

Consent to publish findings of the study was obtained from the participants of the study.

## Data Availability

All the data for this study is available and can be accessed at the KEMRI‐Wellcome Trust Research Programme, subject to institutional data governance policies.
